# Metagenomic and metabolomic profiling of laterally spreading tumors identifies a microbiome with putative pro-tumorigenic features in high-grade intraepithelial neoplasia

**DOI:** 10.3389/fmicb.2026.1844759

**Published:** 2026-09-21

**Authors:** Zhenjiang Wang, Mingyi Guo, Zaiwei Huang, Jianbin Cheng, Yuping Chen, Linmin Lai, Junxian Xiao, Wei Huang, Yuanfei Chen

**Affiliations:** Zhuhai People’s Hospital (The Affiliated Hospital of Beijing Institute of Technology, Zhuhai Clinical Medical College of Jinan University), Zhuhai, China

**Keywords:** high-grade intraepithelial neoplasia, laterally spreading tumors, metagenomic sequencing, sessile serrated lesions, untargeted metabolomic

## Abstract

**Introduction:**

Laterally spreading tumors (LSTs) are pathologically classified into adenomas, including low-grade intraepithelial neoplasia (LGIN) and high-grade intraepithelial neoplasia (HGIN), and sessile serrated lesions (SSL). HGIN has a higher risk of progressing to colorectal cancer.

**Methods:**

This study compared the gut microbiome across these three pathological subtypes using fecal shotgun metagenomic sequencing and non-targeted metabolomics in 53 patients.

**Results:**

Overall community structure was similar among groups by Bray-Curtis NMDS and ANOSIM, although HGIN exhibited higher alpha diversity than SSL and enrichment of inflammation-associated and opportunistic taxa (e.g., Desulfovibrio, Bilophila, Helicobacter, Acinetobacter) alongside depletion of selected commensal taxa associated with mucosal homeostasis, including Bifidobacterium-, Lachnospiraceae-, and Ruminococcus-related species. Functionally, the HGIN-associated microbiome showed an expanded resistome and increased mobile genetic element-related potential, particularly in the HGIN versus LGIN comparison, with enrichment of beta-lactamase genes, MCR-family genes, and mobileOG features related to DNA transfer, recombination, transposition, plasmid maintenance, secretion, pilus-associated functions, and phage-linked mobility. In exploratory analyses, untargeted metabolomics suggested a bile- and lipid-rich metabolic pattern in HGIN, with nominal increases (*P* < 0.05; none significant after FDR correction) in lithocholyltaurine, LysoPE(P-16:0/0:0), tridecanoic acid and cortexolone. GSEA revealed nominal enrichment of unsaturated fatty acid biosynthesis, pyruvate metabolism, and propanoate metabolism. Exploratory species-metabolite correlations linked HGIN-enriched pathobionts with lipid/steroid-, bile-acid-, amino-acid-, and fatty-acid-related metabolites, while HGIN-depleted commensals were associated with amino-acid and organic-acid metabolic features.

**Discussion:**

Together, these findings identify microbial and metabolic features associated with high-grade histopathology within the LST spectrum, including FDR-supported microbial remodeling—pathobiont enrichment and expanded resistome and mobile genetic potential—accompanied by exploratory, nominally significant metabolomic alterations involving bile/lipid- and SCFA-related metabolites.

## Introduction

Laterally spreading tumors (LSTs) are defined as colorectal lesions measuring ≥ 10 mm that grow laterally along the mucosa rather than vertically, and into are histologically classified adenomas, including high-grade intraepithelial neoplasia (HGIN) and low-grade intraepithelial neoplasia (LGIN), and sessile serrated lesions (SSL) (Kudo et al., 2008; Martinelli et al., 2025). Understanding the biology of LSTs is of considerable clinical importance, as these lesions represent 17.2% of advanced colorectal neoplasia, with HGIN demonstrating an incidence of high-grade dysplasia ranging from 20.9% to 33.8% and exhibiting substantial risk for malignant transformation to colorectal cancer (CRC) (Burgess et al., 2017; Saito et al., 2019; Zhao et al., 2014). Patients with LSTs more frequently develop metachronous colorectal neoplasms with high-grade dysplasia or submucosal invasion compared to those with conventional polypoid lesions, with hazard ratios exceeding 2.9 in longitudinal studies (Bogie et al., 2021).

Emerging evidence has established the gut microbiota as an important factor in colorectal carcinogenesis, prompting several investigations into the microbial landscape of LSTs using culture-independent molecular methods (Carding et al., 2015). A recent 16S rRNA gene sequencing study identified fecal *Enterotoxigenic Bacteroides fragilis*, *Peptostreptococcus stomatis*, and *Parvimonas micra* as potential biomarkers for LST diagnosis and adenoma recurrence prediction (Shen et al., 2021). Lin et al. (2025) performed comprehensive fecal metagenomic and serum metabolomic analyses on LST patients, revealing microbial dysbiosis and altered metabolite profiles associated with LST detection (Lin et al., 2025). Kim et al. (2020) reported that fecal untargeted metabolomics in colorectal adenoma patients identified adenoma-associated bioactive lipid signatures, including secondary bile acids and sphingolipids, and demonstrated strong coupling between the gut microbiome and metabolome, supporting the value of metabolomics for investigating microbiota-related metabolic alterations during early colorectal tumorigenesis. Additionally, metagenomics-based investigations have consistently identified microbial signatures enriched in CRC and advanced adenoma, including increased abundance of *Fusobacterium*, *Porphyromonas*, and *Parvimonas*, alongside depletion of *Faecalibacterium* and other commensals (Brennan and Garrett, 2019; Wirbel et al., 2019).

Despite these advances, critical knowledge gaps remain in our understanding of the microbial functional landscape across the LST pathological spectrum. First, existing metagenomic studies of LST have primarily focused on taxonomic profiling (Thomas et al., 2019), with limited exploration of functional gene repertoires—including antibiotic resistance genes (ARGs) and mobile genetic elements (MGEs)—that may mechanistically link microbial communities to adenoma progression. Second, no published study has systematically compared the microbiome functional profiles across HGIN, LGIN, and SSL within the LST spectrum, thereby limiting our ability to identify histological grade-specific microbial contributions to neoplastic transformation.

To address these gaps, we conducted shotgun metagenomic sequencing and untargeted metabolomic analysis of fecal samples from 53 LST patients stratified into HGIN (*n* = 18), LGIN (*n* = 26), and SSL (*n* = 9) groups, followed by comprehensive functional annotation against multiple databases including CARD, KEGG, and mobileOG-db. Our results identify microbial and metabolic features associated with high-grade histopathology within the LST spectrum, characterized by inflammatory adaptation, reduced commensal-associated metabolic homeostasis, enhanced microbial genetic mobility, and altered host-microbe metabolic interactions.

## Materials and methods

### Patients and tissue samples

This study enrolled 53 patients with colorectal adenomas. All patients were explicitly confirmed, at the time of recruitment, to have no antibiotic, probiotic/prebiotic, dietary-supplement, or proton pump inhibitor (PPI) use within the 3–6 months prior to fecal sample collection. None of the participants adhered to a strict vegetarian diet. Two pathologists classified the histological samples as 26 cases of low-grade intraepithelial neoplasia (LGIN), 18 cases of high-grade intraepithelial neoplasia (HGIN), and nine sessile serrated lesions (SSL). Corresponding stool samples underwent metagenomic sequencing and non-targeted metabolomics detection (Figure 1). For all participants, we additionally recorded smoking status, alcohol intake, fasting plasma glucose, blood lipids, diabetes status, metformin use, and other non-antibiotic/non-PPI medication use (Supplementary Table 1). All stool samples were collected before bowel preparation for colonoscopy and stored at −80 °C until processing. The study protocol was approved by the Ethics Committee of Zhuhai People’s Hospital (Zhuhai Hospital Affiliated with Jinan University; Approval number [2022] No. 78). All participants provided written informed consent, and the research adhered to guidelines from China’s National Health Commission.

**FIGURE 1:**
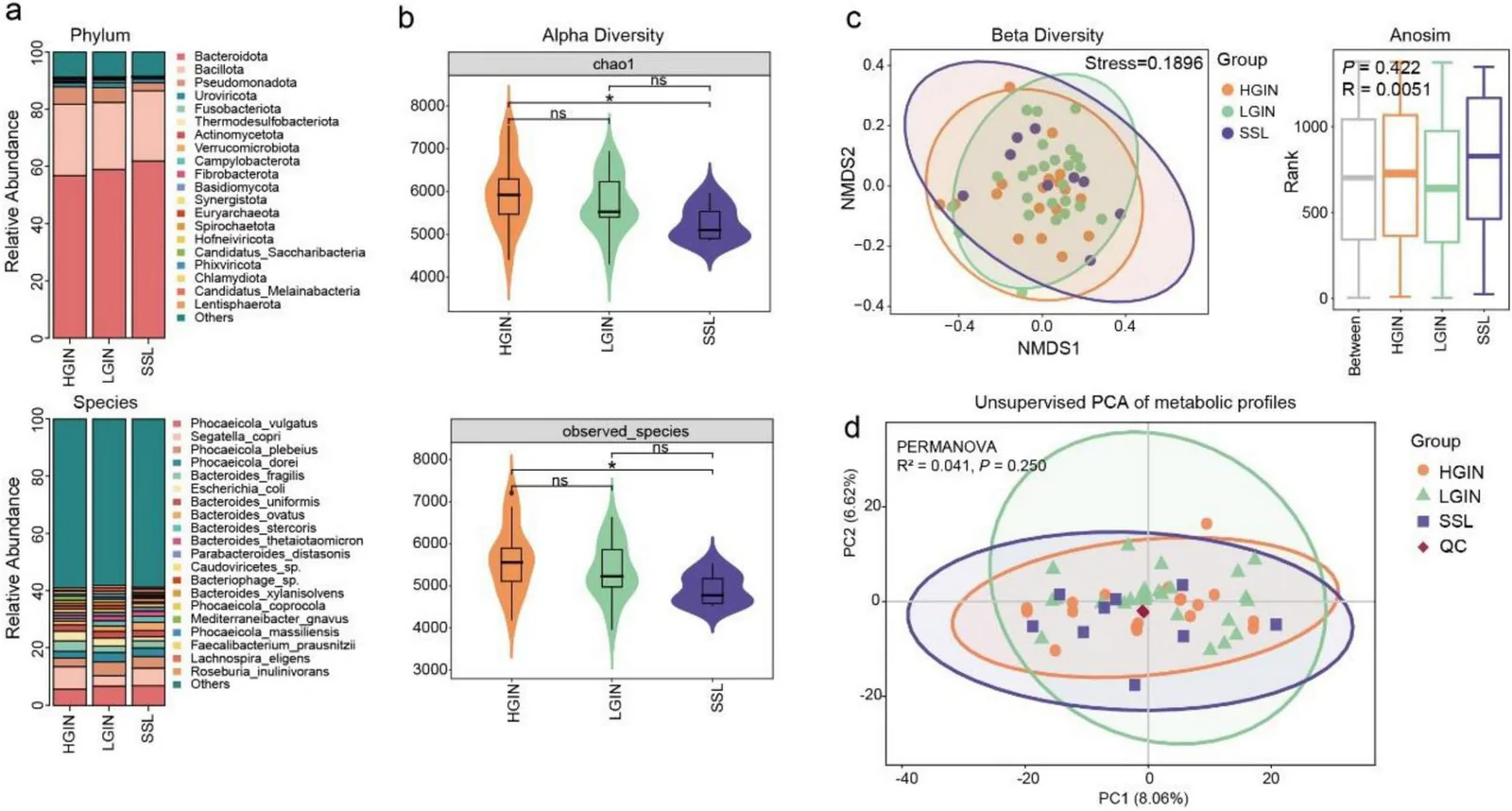
Microbial species composition and metabolic profiles. **(a)** Microbial abundance at the phylum and species levels across high-grade intraepithelial neoplasia (HGIN), low-grade intraepithelial neoplasia (LGIN), and sessile serrated lesions (SSL) groups. **(b)** Alpha diversity of HGIN, LGIN, and SSL groups. **(c)** Beta diversity of HGIN, LGIN, and SSL groups. **(d)** Unsupervised principal component analysis (PCA) of metabolic profiles. Each point represents one biological sample or pooled QC injection; ellipses indicate 95% confidence regions. ns, not significant; *FDR < 0.05.

### DNA extraction, library construction, and sequencing

Total genomic DNA from stool samples was isolated and purified using the pre-packed MagBead Stool gDNA Extraction Kit (AU46111-96, Bioteke) following the manufacturer’s protocol. DNA concentration was quantified with Qubit 1X dsDNA HS Assay Kits (Invitrogen, Q33230). For samples meeting quality criteria, 200 ng DNA was transferred to 0.6 mL low-retention tubes and adjusted to 52 μL with nuclease-free water. Shearing was performed using a Biorupter Pico sonicator (Diagenode) optimized for 200–500 bp fragments. Sheared products underwent bead-based purification with TruSeq reagents, and fragment size distribution was verified via Agilent 2100 Bioanalyzer (High Sensitivity DNA Kit; Agilent, 5067-4627). Samples failing size criteria were re-sheared and re-purified. Fragmented DNA was converted to sequencing libraries using the TruSeq Nano DNA LT Library Prep Kit (Illumina, FC-121-4001). This involved end-repair, adapter ligation, index PCR amplification, and final purification (detailed in kit manual). Library yield was quantified via Qubit 1X dsDNA HS Assay (Invitrogen, Q33230). Qualified libraries underwent paired-end 150 bp sequencing (PE150) on the NovaSeq 6000 platform (Illumina Inc., CA, USA), per standard operating procedures.

### Metagenomic data preprocessing

Raw sequencing reads in FASTQ format underwent quality trimming with fastp (v0.23.4) (Chen, 2025) using the following criteria: adapters were auto-detected and removed, bases with a Phred quality score below Q20 were trimmed using a sliding-window approach, reads shorter than 100 bp after trimming were discarded, and reads with more than 30% ambiguous (N) bases were removed. These thresholds follow widely adopted defaults for shotgun metagenomic preprocessing and were applied uniformly across all samples to ensure comparability (He et al., 2020). Host contamination was depleted by aligning trimmed reads to the human reference genome (hg38) via Bowtie2 (v2.2.0) (Langmead et al., 2018) and retaining only unmapped microbial sequences. Cleaned reads from each sample were de novo assembled into contigs (>500 bp) using MEGAHIT (v1.2.9) (Li et al., 2015), yielding FASTA files. Coding sequences (CDS) were predicted with MetaGeneMark (v3.26) (Lomsadze et al., 2021), filtering out predictions shorter than 100 bp. Non-redundant unigenes were generated from CDS via MMseqs2 clustering (v15-6f452) (Mirdita et al., 2021) at 95% identity and 90% coverage, selecting the longest representative sequence per cluster. Sample-specific read counts per unigene were quantified by Bowtie2 mapping, excluding unigenes with ≤2 reads across all samples. Unigene protein sequences received taxonomic annotations against the NR_meta database using DIAMOND (v0.9.14) (Buchfink et al., 2021), retaining best-hit alignments with an e-value threshold of <1e-5. For unigenes with multiple equally scoring best hits, taxonomic assignment was resolved using a lowest-common-ancestor approach to avoid over-specific misclassification. Species-level features were required to be detected in at least 10% of samples to be retained for downstream differential abundance testing. Functional profiles were assigned via DIAMOND searches against KEGG (v87.1), eggNOG (v5.0), CAZy (v2022-08-06) (Drula et al., 2021), CARD (v3.2.5), VFDB (v2023-03-03) (Liu et al., 2022), and mobileOG-db (Brown et al., 2022). Species- and function-level abundances were derived from unigene quantification profiles.

All libraries were prepared with the same kit lot and sequenced together in a single run on one NovaSeq 6000 flow cell, minimizing technical batch effects; no cross-batch correction was therefore required. The unfiltered raw count species abundance table was used for the calculation of species diversity indices, including alpha and beta diversity metrics. To control for differences in sequencing depth across samples, species-level alpha diversity—including Chao1 and observed species richness—was computed following rarefaction to the minimum sample read depth using QIIME1 (Caporaso et al., 2010), and rarefaction curves were plotted in R (v3.6.0) to confirm that diversity estimates had reached saturation, thereby minimizing confounding by uneven library size. Beta-diversity was quantified using Bray-Curtis dissimilarities and visualized by non-metric multidimensional scaling (NMDS), with overall group patterns initially assessed using ANOSIM in R. To formally evaluate beta-diversity differences and the contribution of clinical covariates, we additionally applied PERMANOVA to the Bray-Curtis distance matrices, modeling histological group as the main factor and including age, sex, smoking, alcohol, and diabetes as covariates where indicated, thereby estimating both statistical significance and the variance explained (R^2^) by each term. To assess whether host-related factors were balanced across pathological groups, continuous covariates (fasting glucose and lipids) were compared using the Kruskal–Wallis test, and categorical covariates (smoking, alcohol, diabetes, and metformin use) using Fisher’s exact test. Prior to differential testing, raw counts were normalized using the cumulative sum scaling (CSS) method implemented in metagenomeSeq (v1.38.0) (Paulson et al., 2013), which corrects for uneven sequencing depth and library size and mitigates the compositional bias introduced by preponderant features. Then, pairwise species- and function-level differential abundances between groups were evaluated using the metagenomeSeq, with age and sex included as covariates in the statistical model. Multiple testing correction was conducted using the built-in Benjamini–Hochberg (BH) procedure in metagenomeSeq to control the false discovery rate (FDR). Features with an FDR < 0.05 were considered statistically significant. For all species- and function-level comparisons, effect estimates were reported as log_2_ fold changes (log_2_FC). To facilitate interpretation of effect magnitude, log_2_FC were categorized into predefined magnitude bins based on their absolute value: negligible (| log_2FC| < 0.5; small (0.5 ≤ | log_2FC| < 1), moderate (1 ≤ | log_2FC| < 2), and large (| log_2FC| ≥ 2). Given the limited SSL sample size, HGIN-versus-SSL results were interpreted as exploratory even when they met the FDR threshold. Differential species or functional profiles were visualized using R.

### Metabolite extraction and UPLC-HRMS analysis

A total of 50 mg (±5 mg) of each stool sample was extracted with 500 μL of prechilled 80% methanol, homogenized with steel beads, incubated at −20 °C for 30 min for protein precipitation, and centrifuged at 20,000 *g* for 10 min at 4 °C. The supernatant was collected, centrifuged again for 5 min, and transferred to a fresh vial for UPLC-HRMS analysis. QC samples were prepared by pooling equal aliquots of the supernatants from all samples. Chromatographic separation was performed on an ACQUITY UPLC HSS T3 column (100 × 2.1 mm, 1.8 μm; Waters) using water containing 5 mmol/L ammonium acetate and 5 mmol/L acetic acid as mobile phase A and acetonitrile as mobile phase B. The flow rate was 0.35 mL/min, the injection volume was 4 μL, and the column temperature was maintained at 40 °C. Mass spectrometric detection was conducted on a Q Exactive Plus high-resolution tandem mass spectrometer (Thermo Fisher Scientific) in both positive and negative electrospray ionization modes. Data were acquired in full-scan and data-dependent acquisition modes over an m/z range of 70-1050, with the top five most intense ions selected for MS/MS analysis and a dynamic exclusion time of 6 s.

### Metabolomics data processing and statistical analysis

Raw LC-MS data were converted to mzXML format and processed using the XCMS (Smith et al., 2006), CAMERA (Kuhl et al., 2012), and metaX packages (Wen et al., 2017) in R for peak detection, alignment, retention time correction, secondary peak grouping, and isotope/adduct annotation. Metabolite features were defined by retention time and m/z, and a three-dimensional data matrix containing feature identifiers, sample information, and ion intensities was generated for downstream analysis. Putative metabolite annotation was performed by matching accurate mass data against the KEGG and HMDB databases (Wishart et al., 2022), using a mass tolerance of less than 10 ppm; candidate annotations were further supported by isotopic distribution patterns and validated with an in-house MS/MS spectral library.

Statistical analyses were conducted in R. Metabolite data processing included data filtering, imputation, and normalization. Features were filtered out if missing values exceeded 80% across samples or 50% in QC samples. Missing values were imputed using the K-nearest neighbors (KNN) method, and data normalization was performed using the probabilistic quotient normalization (PQN) method. Differential metabolite analyses were performed with R using Wilcoxon test. PLS-DA models were built with ropls using 7-fold cross-validation and 200 response permutations; model quality was assessed by R^2^Y, Q^2^, permutation *P* and permutation intercepts. Variable importance in projection (VIP) values were calculated for each feature. Unsupervised principal component analysis (PCA) was conducted in R using the stats package to visualize overall metabolic profiles, including pooled QC injections. To statistically assess global differences in metabolomic composition among HGIN, LGIN, and SSL groups, PERMANOVA was performed using the vegan R package with 9,999 permutations. Homogeneity of multivariate dispersion among groups was assessed using vegan package. Exploratory differential metabolites were defined by *P* < 0.05, fold change > 1.2 (effect estimates), and VIP ≥ 1. After Benjamini-Hochberg FDR correction, no metabolites had an FDR < 0.05. Accordingly, all metabolite-level findings reported in this study are exploratory and hypothesis-generating rather than statistically significant after correction for multiple testing. Effect magnitude was similarly summarized using predefined bins based on fold change: small (1.2–2-fold), moderate (2–4-fold), and large (≥4-fold). Metabolite set enrichment analysis was conducted using GSEA (v4.1.0) (Subramanian et al., 2005), and pathways with | NES| > 1, nominal *P* < 0.05, and FDR < 0.25 were regarded as nominally enriched.

To integrate the metagenomic and metabolomic layers, we performed Spearman rank correlation analyses between the relative abundances of selected differentially abundant taxa and selected key differential metabolites. Due to the small sample size, all correlations had FDR greater than 0.05 after global multiple-testing correction. Therefore, nominal *P* < 0.05 and | ρ| ≥ 0.4 were used to identify potentially meaningful correlations, which were considered exploratory findings only.

## Results

### Sample collection and sequencing performance

A total of 53 fecal samples were collected from adenoma patients for metagenomic sequencing analysis. Patients ranged from 25 to 80 years of age (mean: 57.1 years), with 45.28% female participants. Lesions were distributed across multiple colonic sites, with the ascending colon being the most frequently sampled region. Pathologically, samples were stratified into three groups: high-grade intraepithelial neoplasia (HGIN, *n* = 18), low-grade intraepithelial neoplasia (LGIN, *n* = 26), and sessile serrated lesions (SSL, *n* = 9), primarily characterized as tubulovillous or tubular adenomas (Supplementary Table 1). Host-related factors that may influence gut microbial composition-including smoking, alcohol intake, diabetes status, metformin use, and other non-antibiotic/non-PPI medications-did not differ significantly across HGIN, LGIN, and SSL (*P* > 0.05). No participant used probiotics, prebiotics, or dietary supplements.

Metagenomic sequencing generated an average of 6.20 Gb per sample, with a mean effective read ratio of 96.34% after quality control and host genome decontamination. All samples demonstrated high base quality, with Q20 and Q30 scores exceeding 97.50% and 92.51%, respectively, and minimal host genome contamination (mean: 0.04%; Supplementary Table 2). To assess whether sequencing depth was sufficient and comparable across samples, rarefaction curves were generated for species-level alpha diversity metrics (Chao1), confirming that diversity estimates approached saturation at the achieved sequencing depth across all three groups (Supplementary Table 3). To obtain a more direct functional readout of the gut microbiota, we performed untargeted metabolomic analysis on the same samples. A total of 1,801 metabolites were identified in the untargeted metabolomics analysis (Supplementary Table 4). HMDB annotation showed that the identified metabolites were mainly classified as lipids and lipid-like molecules (*n* = 441) and organoheterocyclic compounds (*n* = 377), while KEGG annotation indicated that they were primarily enriched in pathways related to ABC transporters (*n* = 52), biosynthesis of secondary metabolites (*n* = 166), biosynthesis of plant secondary metabolites (*n* = 57), biosynthesis of cofactors (*n* = 45), and biosynthesis of amino acids (*n* = 37).

### Similar microbial community structure and metabolic profiles despite differential alpha diversity

At the phylum level, all three groups were dominated by Bacteroidota (56.81%–61.91%) and Bacillota (23.45-24.92%). At the species level, *Phocaeicola vulgatus*, *Segatella copri*, and *Phocaeicola plebeius* were dominant across all groups. HGIN samples were enriched in *Segatella copri* (7.92%) and *Bacteroides fragilis* (3.71%) compared to LGIN and SSL (Figure 1a). Alpha diversity metrics revealed significant differences between HGIN and SSL groups in both observed species richness (FDR = 0.04) and Chao1 estimates (FDR = 0.03), with HGIN samples exhibiting higher richness compared to SSL samples (Figure 1b). Beta diversity analysis based on Bray-Curtis dissimilarity revealed no significant differences in microbial community structure between groups. Covariate-adjusted PERMANOVA further confirmed that histological group was not significantly associated with overall community structure after adjustment for age, sex, smoking, alcohol, and diabetes (Supplementary Table 5). Overall, the three groups showed similar community structures as demonstrated by NMDS ordination analysis (ANOSIM, R = 0.01, *P* = 0.42), indicating that the microbial composition patterns were not significantly stratified by lesion pathology type (Figure 1c).

Similarly, unsupervised PCA of MS/MS-identified metabolites showed substantial overlap among samples from HGIN, LGIN and SSL (Figure 1d). The tight clustering of pooled QC injections supported analytical stability. PERMANOVA did not identify a significant difference in overall metabolic composition among the three biological groups (R^2^ = 0.041, *P* = 0.250), and group dispersions were comparable (PERMDISP *P* = 0.622).

### HGIN-associated microbiota shifts toward inflammation-adapted and opportunistic taxa

After adjustment for age and sex, metagenomeSeq identified differentially abundant species between HGIN and SSL, as well as between HGIN and LGIN. Relative to SSL, HGIN displayed elevated abundance in 96 species and reduced abundance in 82 species after BH correction (FDR < 0.05). In the HGIN versus LGIN comparison, 87 species were enriched and 81 species were depleted in HGIN at FDR < 0.05 (Figure 2a and Supplementary Table 6). These comparisons revealed that HGIN-associated microbial communities showed enrichment of inflammatory and pathobiont-related taxa with concomitant depletion of commensal taxa linked to mucosal homeostasis (Figure 2b). Specifically, inflammation-associated taxa enriched in HGIN included *Desulfovibrio* sp. (log_2_FC = 2.97 vs. SSL, large; 1.45 vs. LGIN, moderate), *Helicobacter pylori* (log_2_FC = 1.290 vs. LGIN, moderate). Enterococcus avium (log_2_FC = 0.363 vs. LGIN) and *Bilophila unclassified* (log_2_FC = 0.332 vs. SSL) were also enriched but with negligible effect magnitude. Pathobiont- or opportunistic pathogen-associated taxa included *Citrobacter sp. wls716* (log_2_FC = 5.25 vs. LGIN, large), *Acinetobacter sp. YH12144* (log_2_FC = 2.35 vs. LGIN, large). In contrast, taxa with putative commensal, barrier-supportive, or short-chain fatty acid (SCFA)-associated functions, including *Bifidobacterium spp.* (log_2_FC = −3.22 vs. LGIN, large), *Lachnospiraceae bacterium* spp. (log_2_FC = −0.22 and −0.24 vs. LGIN, small), and multiple Ruminococcus-related species or strains (log_2_FC = −2.27∼−1.51 vs. SSL, large to moderate), were depleted in HGIN.

**FIGURE 2:**
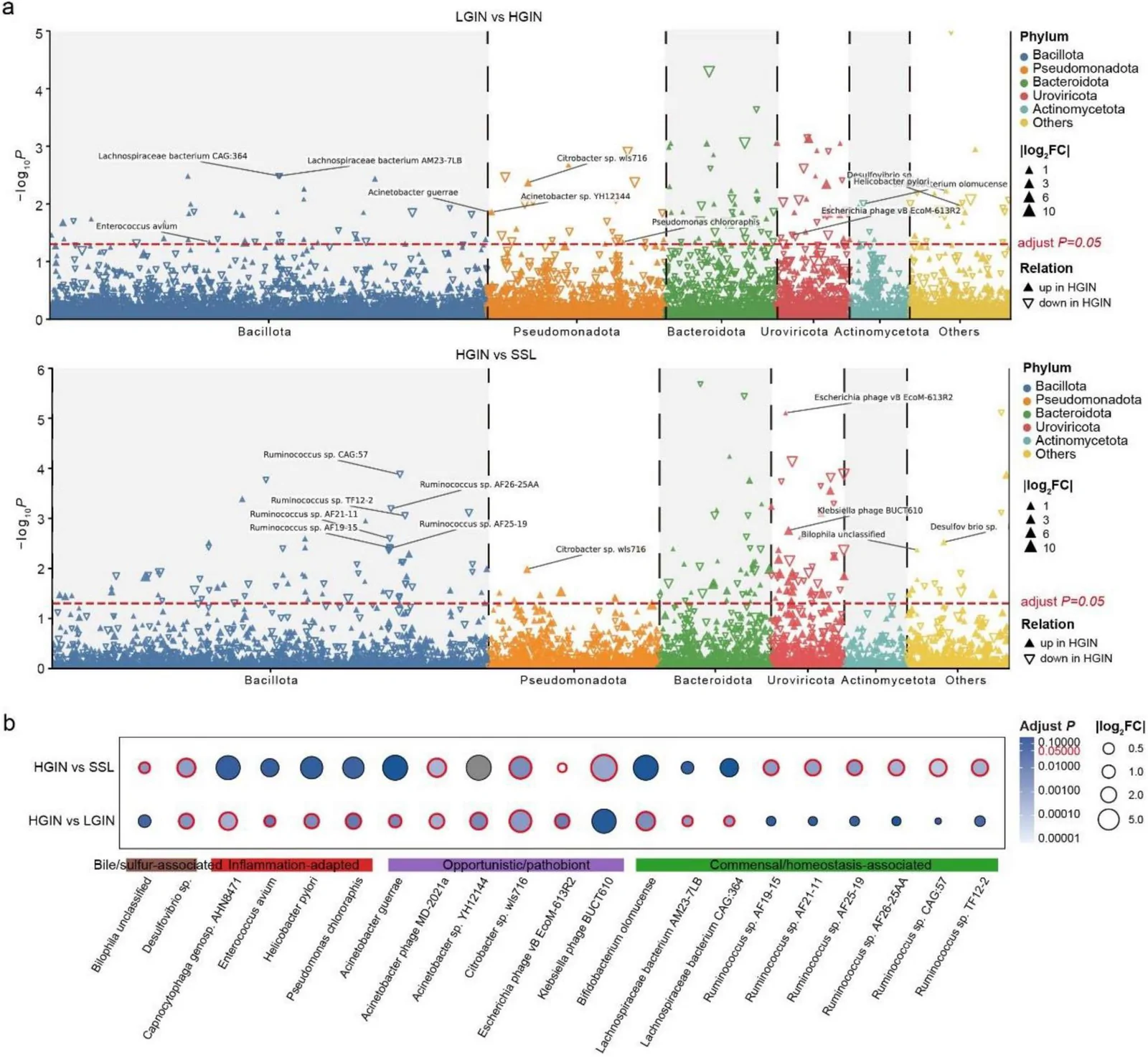
High-grade intraepithelial neoplasia (HGIN)-associated species-level microbial alterations. **(a)** Manhattan plots showing differentially abundant species between HGIN and sessile serrated lesions (SSL) groups, and between HGIN and low-grade intraepithelial neoplasia (LGIN) groups, calculated using metagenomeSeq with age and sex included as covariates. The red dashed line indicates the adjusted *P*-value threshold of 0.05. Species are colored by phylum, with the five most abundant phyla shown individually and the remaining phyla grouped as “Others.” Upward filled triangles indicate species enriched in HGIN, whereas downward open triangles indicate species depleted in HGIN. Triangle size represents the absolute log_2_ fold change. **(b)** Bubble plot of differentially abundant species. Colored rectangles at the bottom indicate bacteria with different functional characteristics. Bubble size represents | log_2_FC|, and bubble color represents FDR. Bubbles with red outlines indicate FDR < 0.05.

### HGIN-associated microbiota shows enhanced resistome and mobile genetic potential

To characterize the functional gene potential associated with the HGIN species profile, we annotated and analyzed antibiotic resistance genes (ARGs). MetagenomeSeq analysis revealed that the HGIN-associated microbiota contained a stronger resistome signal, particularly in the HGIN versus LGIN comparison (Figure 3a and Supplementary Table 7). Twenty-one ARGs were significant (FDR < 0.05), of which 15 showed higher abundance in HGIN. The enriched beta-lactam resistance module comprised genes spanning a broad range of effect magnitudes, from large enrichment of CMY-65 (log_2_FC = 3.52) and GOB-16 (log_2_FC = 2.57) beta-lactamase genes to moderate enrichment of PER-3 (log_2_FC = 1.69) and small enrichment of CARB-6 (log_2_FC = 0.81), together suggesting an increased capacity to tolerate beta-lactam pressure that may facilitate persistence of pathobiont-associated bacterial groups under antimicrobial or ecological stress. In addition, the polymyxin and host antimicrobial peptide-related module also enriched in HGIN, represented by MCR-family genes with moderate effect sizes (MCR-3.8: log_2_FC = 1.64; MCR-4.4: log_2_FC = 1.12). Because MCR enzymes modify lipid A, this module may reflect enhanced outer-membrane remodeling in Gram-negative bacteria and improved resistance to cationic antimicrobial pressures.

**FIGURE 3:**
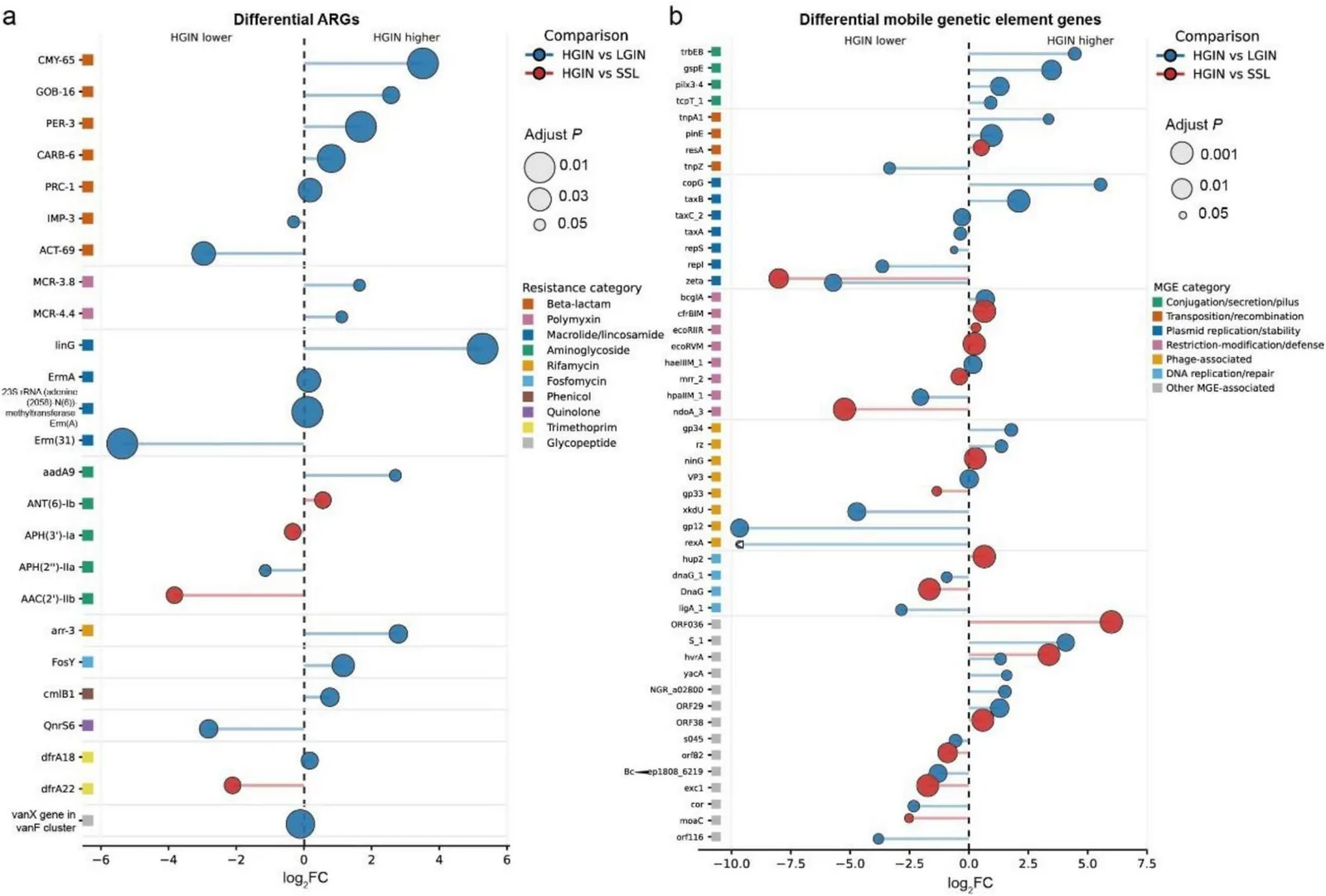
Differential antibiotic resistance genes and mobile genetic element-associated genes in high-grade intraepithelial neoplasia (HGIN). Differentially abundant antibiotic resistance genes **(a)** and mobile genetic elements **(b)** between HGIN and sessile serrated lesions (SSL) groups, and between HGIN and low-grade intraepithelial neoplasia (LGIN) groups. The x-axis shows log_2_FC for HGIN relative to the reference group; values to the right of zero indicate higher abundance in HGIN, whereas values to the left indicate lower abundance in HGIN. Bubble color denotes the comparison, and bubble size is inversely proportional to the FDR, with larger bubbles indicating smaller FDR.

The resistome signal was accompanied by increased mobile genetic element-related features. We characterized bacterial mobile genetic elements (MGEs) in the HGIN group using the mobileOG-db database. In HGIN versus LGIN, 34 mobileOG features were significant (FDR < 0.05), with 18 enriched in HGIN (Figure 3b and Supplementary Table 8). The enriched features were related to DNA transfer, recombination, transposition, plasmid maintenance, secretion, pilus-associated functions, or phage-linked mobility, including taxB (log_2_FC = 2.10), pinE (log_2_FC = 0.96), gspE (log_2_FC = 3.48), pilx3-4 (log_2_FC = 1.30), tnpA1 (log_2_FC = 3.36), copG (log_2_FC = 5.54), and hvrA (log_2_FC = 1.33). In the exploratory HGIN versus SSL, 17 features were significant (FDR < 0.05), with nine enriched in HGIN. Overlapping or related mobileOG signals included hvrA (log_2_FC = 3.37), hup2 (log_2_FC = 0.65), ecoRVM (log_2_FC = 0.23), cfrBIM (log_2_FC = 0.66), ninG (log_2_FC = 0.27), and ecoRIIR (log_2_FC = 0.30).

### Exploratory metabolomic differences in HGIN involve bile-acid- and lipid-related features

Untargeted metabolomic profiling further suggested that HGIN was nominally associated with a pro-inflammatory microbial milieu. Among bile-acid, lipid, and steroid-related metabolites nominally elevated in HGIN versus SSL (*P* < 0.05), cortexolone showed the largest nominal fold-change (FC = 28.76), followed by moderate nominal increases in LysoPE(P-16:0/0:0) (FC = 2.61) and tridecanoic acid (FC = 2.27), and a small nominal increase in lithocholyltaurine (FC = 1.23). Because none of these metabolites remained significant after multiple-testing correction, these findings were considered exploratory. Taurine-conjugated bile acids have been reported to promote pathobiont expansion and colitis in prior mechanistic studies, and lysophosphatidylethanolamine species are characteristic membrane phospholipids of Gram-negative bacteria; based on this existing literature, the nominal elevation of these metabolites in HGIN may be consistent with a gut environment favoring bile-tolerant, inflammation-associated opportunistic pathogens (Figure 4a and Supplementary Table 9).

**FIGURE 4:**
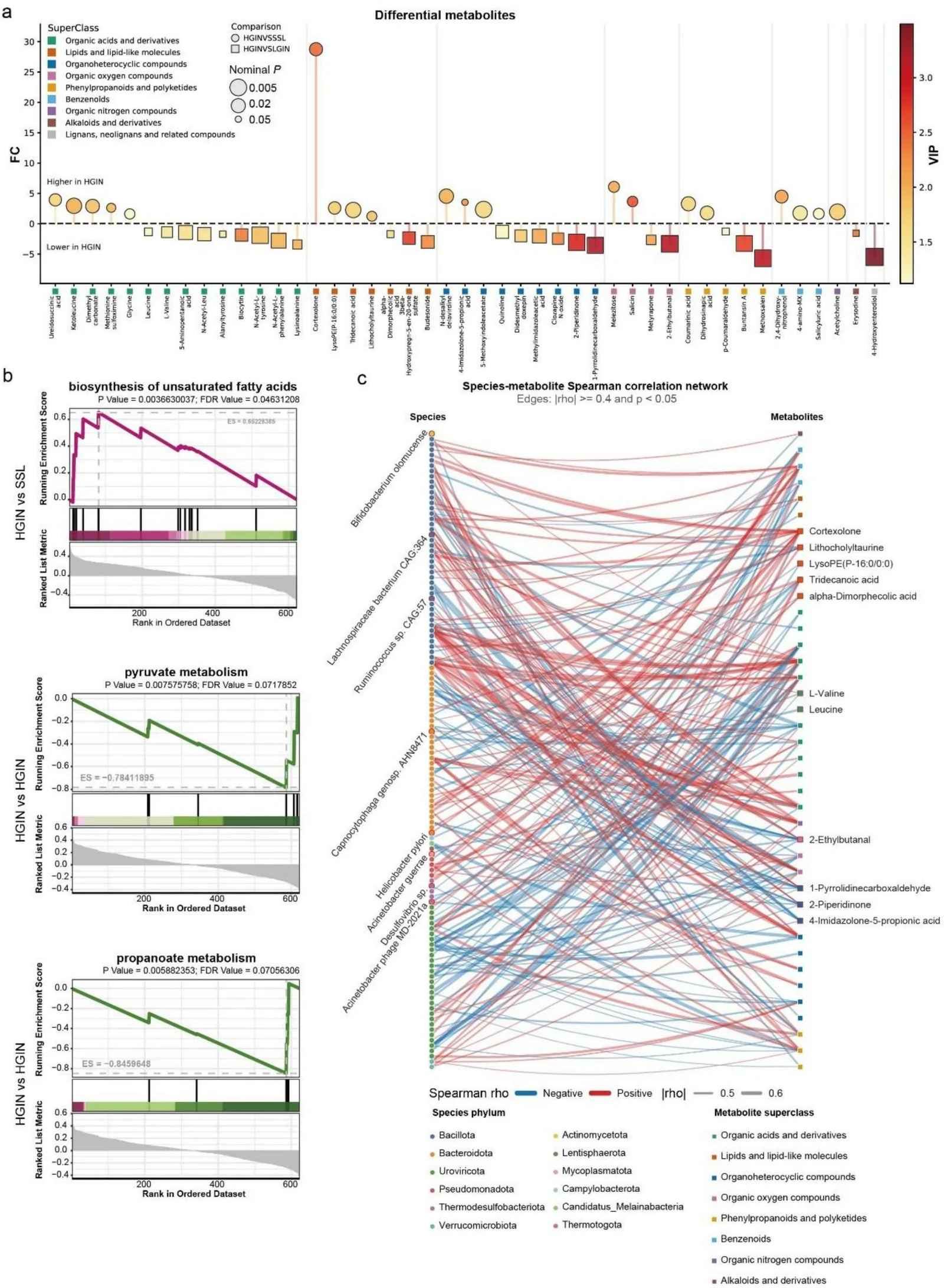
Metabolic remodeling and species-metabolite associations in high-grade intraepithelial neoplasia (HGIN). **(a)** Lollipop bubble plot of nominally differential metabolites (*P* < 0.05, FC > 1.2, VIP ≥ 1) in HGIN vs. low-grade intraepithelial neoplasia (LGIN) and HGIN vs. sessile serrated lesions (SSL) comparisons. The y-axis shows signed fold changes (FC) values, where positive values indicate higher abundance in HGIN and negative values indicate lower abundance in HGIN. Circles represent HGIN vs. SSL results, and squares represent HGIN vs. LGIN results. Bubble color indicates variable importance in (VIP) score, and bubble size is inversely proportional to the nominal *P*-value. None of these metabolites remained significant after Benjamini–Hochberg false discovery rate (FDR) correction; findings are shown for exploratory and hypothesis-generating purposes only. **(b)** GSEA analysis of gut microbiota metabolites. **(c)** Exploratory Spearman correlation network linking differential species and differential metabolites. Correlations with | rho| ≥ 0.4 and nominal *P* < 0.05 are shown. Species nodes are colored by phylum, metabolite nodes are colored by metabolite Superclass, and edge color indicates correlation direction, with red denoting positive correlations and blue denoting negative correlations. Edge width represents the absolute Spearman correlation coefficient.

To further characterize the metabolic landscape of HGIN, we performed GSEA on untargeted fecal metabolomics data from the HGIN-versus-LGIN and HGIN-versus-SSL comparisons. In the exploratory HGIN vs. SSL comparison, biosynthesis of unsaturated fatty acids was nominally enriched in HGIN (NES = 1.87, *P* = 0.004, FDR < 0.25; Figure 4b). In the HGIN vs. LGIN comparison, pyruvate metabolism (NES = 1.74, *P* = 0.008, FDR < 0.25) and propanoate metabolism (NES = 1.75, *P* = 0.006, FDR < 0.25) were enriched in HGIN. Together, these findings may suggest that HGIN is associated with metabolic remodeling involving lipid metabolism and short-chain fatty acid-associated pathways (Figure 4b).

### Exploratory correlations between HGIN-associated taxa and metabolites

Exploratory Spearman correlation analysis was used to examine potential links between HGIN-associated taxa and metabolites (Figure 4c and Supplementary Table 10). None of the correlations remained significant after global FDR correction; the nominal associations below are therefore reported solely as hypothesis-generating patterns. Among HGIN-enriched inflammation-adapted or opportunistic taxa, Desulfovibrio_sp. was negatively correlated with the lipid/steroid-related metabolite 3β-hydroxypregn-5-en-20-one sulfate (Spearman’s ρ = −0.412, *P* = 0.005). *Helicobacter pylori* was negatively correlated with the fatty-acid derivative alpha-dimorphecolic acid (ρ = −0.438, *P* = 0.003). In the HGIN versus SSL comparison, *Acinetobacter phage MD-2021a* was positively correlated with the bile-acid-related metabolite lithocholyltaurine (ρ = 0.480, *P* = 0.011). In contrast, HGIN-depleted commensal taxa were associated with metabolites involved in amino-acid, organic-acid, and related metabolic processes: *Lachnospiraceae bacterium CAG:364* was positively correlated with 2-ethylbutanal (ρ = 0.452, *P* = 0.002), 2-piperidinone (ρ = 0.423, *P* = 0.004), L-valine (ρ = 0.412, *P* = 0.006), and 1-pyrrolidinecarboxaldehyde (ρ = 0.409, *P* = 0.006), whereas *Ruminococcus sp. CAG:57* was negatively correlated with the histidine-metabolism-related metabolite 4-imidazolone-5-propionic acid (ρ = −0.429, *P* = 0.026). Because none of these correlations survived global FDR correction, they do not establish a coordinated microbiota–metabolite axis. Instead, they provide hypothesis-generating patterns which potentially linked inflammation-adapted microbial enrichment, loss of commensal-associated metabolic homeostasis, and metabolic conditions favorable for malignant progression. It may guide targeted validation of microbiota–metabolite relationships in larger cohorts.

## Discussion

The present study employed shotgun metagenomic sequencing together with untargeted fecal metabolomics to characterize the gut microbial and metabolic landscape associated with HGIN, LGIN, and SSL within the LST spectrum, with particular emphasis on functional gene repertoires that may be associated with high-grade histopathology. Taxonomic shifts in HGIN included enrichment of inflammation-associated and opportunistic pathogenic taxa alongside depletion of SCFA-producing commensals. These metagenomic changes were accompanied by metabolomic alterations consistent with a pro-inflammatory intestinal environment, including nominally increased lithocholyltaurine and phospholipid-related metabolites, and enrichment of pathways related to unsaturated fatty acid biosynthesis, pyruvate metabolism, and propanoate metabolism. Our multi-layer functional annotations (ARGs and MGEs) and metabolomic pathways converge on a consistent theme: the HGIN microbiome is enriched for traits that could plausibly favor persistence of opportunistic taxa, increased host–microbe contact, and enhanced host stress responses, even when global beta-diversity separation is limited. The evidentiary strength differed across analyses: FDR-supported metagenomic findings from the HGIN-versus-LGIN comparison constitute the more stable within-cohort observations, whereas HGIN-versus-SSL and nominal metabolomic or correlation findings should be interpreted as exploratory.

Our integrated metagenomic and metabolomic analysis of fecal samples from adenoma patients stratified by lesion pathology revealed that, despite overlapping overall microbial community structure and metabolic profile as assessed by unsupervised ordination, HGIN was associated with several specific, FDR-supported microbial and exploratory metabolomic features distinguishing it from LGIN and SSL. The HGIN-associated microbiota exhibited a clear shift toward opportunistic and inflammation-associated taxa—including *Desulfovibrio* sp., and *Bilophila unclassified*—with a concomitant depletion of commensal genera such as *Bifidobacterium*, *Lachnospiraceae*, and *Ruminococcus*. These findings are broadly consistent with prior reports that enrichment of pathobionts, alongside the loss of SCFA-producing commensals, is a recurring feature of the adenoma-carcinoma sequence. The consistent enrichment of *Desulfovibrio* sp. is particularly relevant because sulfate-reducing bacteria can generate hydrogen sulfide, a microbial metabolite implicated in epithelial stress, mucosal inflammation, oxidative damage, and DNA damage (Attene-Ramos et al., 2006; Yazici et al., 2017). The increase in *Bilophila_unclassified* in HGIN further supports the presence of a bile- and sulfur-associated inflammatory niche, as bile acid-driven expansion of *Bilophila* has been linked to intestinal inflammation in experimental models (Devkota et al., 2012). Such sulfate-reducing and bile-tolerant bacteria may contribute to a mucosal environment enriched in hydrogen sulfide, secondary bile-acid stress, oxidative stress, and inflammatory signaling. Based on prior mechanistic literature, these associations could plausibly impair epithelial barrier integrity, promote epithelial stress responses, and increase the likelihood that dysplastic epithelial cells acquire or maintain malignant traits (Attene-Ramos et al., 2006; Devkota et al., 2012; Yazici et al., 2017). Because our data derive from shotgun metagenomics, these remain hypotheses regarding genetic and metabolic potential in our cohort. Metabolomic profiling offered exploratory support for this pro-inflammatory pattern: the nominal increased lithocholyltaurine and lysophosphatidylethanolamine species in HGIN are consistent with the notion that taurine-conjugated bile acids can promote the expansion of bile-tolerant pathobionts and contribute to colitis-associated inflammation, while phosphatidylethanolamine and lysophospholipid species are well-established membrane constituents of Gram-negative bacteria and may reflect their increased abundance (Cai et al., 2022; Ridlon et al., 2016; Zheng et al., 2017). Moreover, the nominal elevation of bile-acid and lipid-related metabolites in HGIN is consistent with previous evidence linking bile acid dysregulation, microbial bile acid transformation, epithelial stress, and colorectal carcinogenesis (Ajouz et al., 2014; Jia et al., 2018). Conversely, depletion of Bifidobacterium, Lachnospiraceae, and Ruminococcus may indicate loss of commensal functions related to fermentation, short-chain fatty acid production, epithelial barrier maintenance, and anti-inflammatory immune tone (Furusawa et al., 2013; Louis et al., 2014).

At the functional level, the HGIN microbiome was characterized by a substantially expanded repertoire of antibiotic resistance genes and mobile genetic elements. Beta-lactamase enrichment may allow opportunistic taxa to persist under cell-wall-targeting antimicrobial pressure, whereas MCR-family genes encode lipid A modification systems that could increase tolerance to polymyxins and possibly host antimicrobial peptides (Liu et al., 2016; Munita and Arias, 2016). At the community level, the expanded genetic repertoire for antimicrobial resistance and horizontal gene transfer detected in HGIN may reflect an increased potential to sustain inflammation-adapted pathobionts and facilitate the spread of adaptive traits within the microbial community (Allen et al., 2010). Such persistence may prolong epithelial exposure to microbial products, inflammatory stimuli, and metabolically stressful conditions, thereby creating a permissive environment for high-grade dysplasia and subsequent malignant transformation (Arthur et al., 2012). However, whether these genes are actively expressed and translate into functional phenotypes *in vivo* remains to be confirmed by metatranscriptomic or metaproteomic validation.

These findings, although exploratory, have several potential translational implications for the management of laterally spreading tumors. First, the HGIN-associated taxonomic and functional signatures—particularly the enrichment of inflammation-adapted pathobionts, an expanded resistome, and bile/lipid-related metabolites—could serve as candidate non-invasive fecal markers to complement endoscopic and histological assessment in identifying lesions with higher malignant potential; because stool is collected non-invasively and prior to bowel preparation in our workflow, such markers could be readily embedded in existing screening pathways. Second, a composite microbial–metabolic signature could contribute to risk stratification, helping to prioritize patients whose LGIN or SSL lesions carry HGIN-like microbial features for closer evaluation. Future studies with larger, independent cohorts may benefit from integrative multimodal analyses combining microbiome, metabolomic, clinical, and imaging features to improve risk stratification and mechanistic understanding of serrated pathway lesions (He et al., 2026; Zhang et al., 2025). We emphasize that these directions are hypotheses generated by an exploratory, single-center study without a healthy comparator, and would require validation in prospective, adequately powered, ideally multi-center cohorts before any clinical application.

Several limitations should be considered when interpreting these findings. First, functional inference is based on metagenomic annotation and differential abundance testing; while it provides strong hypotheses, it does not by itself confirm expression, activity, or host impact of the implicated pathways (e.g., actual mucus degradation rates, inflammatory signaling, or horizontal transfer events), which would require orthogonal validation such as metatranscriptomics and metaproteomics to confirm expression and translation, targeted quantitative metabolomics (e.g., SCFAs and bile acids), culture-based assays, strain-level resolution of ARG/MGE carriage, and experimental models (e.g., gnotobiotic or organoid systems) to test causal contributions. Accordingly, all associations reported here should be interpreted as microbial genetic and metabolic potential associated with pathological grade rather than as functionally demonstrated drivers of progression. Multimodal frameworks that integrate microbial, transcriptomic and histopathological layers for cancer stratification (He et al., 2026) indicate how such complementary data can be combined to strengthen the functional and clinical interpretation of microbiome associations. In addition, because ARG and mobile genetic element abundances were derived from unigene-level annotation rather than contig- or genome-resolved analysis, we cannot confirm genomic co-localization between these functional elements and the specific taxa identified as differentially abundant; all ARG/MGE findings should therefore be interpreted as reflecting community-level functional potential rather than confirmed genetic linkage to particular pathobiont species. Strain-resolved approaches such as metagenome-assembled genome (MAG) binning or long-read sequencing would be required to establish such linkage in future studies. Second, the SSL subgroup comprised only nine patients, which substantially limits the statistical power of HGIN vs. SSL pairwise comparisons. Minimum detectable effect size (sensitivity) analysis (two-sided Wilcoxon rank-sum test, α = 0.05, power = 0.80) indicates that, with *n* = 9 vs. *n* = 18, only large effect sizes (Cohen’s d = 1.19) are reliably detectable. Accordingly, HGIN vs. SSL comparisons should be interpreted as exploratory. Independent validation in a larger, multi-center cohort with matched HGIN/LGIN/SSL stratification will be required to confirm their generalizability and clinical utility before they can be considered candidate biomarkers. Third, our study did not include a healthy control group, as all participants had laterally spreading tumors undergoing colonoscopy and fecal sampling. Consequently, we cannot determine from these data alone whether the baseline microbiome and metabolome of LST patients are already dysbiotic compared with the general population. Our findings should therefore be interpreted as histological grade-specific differences within the LST spectrum (HGIN, LGIN, SSL), rather than as absolute disease markers of LST versus health. Integrating our signatures with large-scale case–control metagenomic resources and prospective cohorts including healthy individuals will be necessary to establish their performance as diagnostic or risk-stratification biomarkers. Fourth, after Benjamini–Hochberg correction, no individual metabolite remained statistically significant (all FDR > 0.05). We therefore emphasize that all metabolite-level findings, including the bile-acid, lipid, and steroid-related signatures discussed above, represent nominal, exploratory associations rather than confirmed differential metabolites, and should be interpreted with appropriate caution pending validation using targeted, quantitative metabolomic assays in larger, independent cohorts. In addition, although age and sex were available and incorporated into post-hoc covariate-adjusted analyses, BMI and detailed dietary patterns were not systematically recorded and could only be indirectly approximated by the exclusion of strict vegetarians. Residual confounding by adiposity, long-term diet or unmeasured lifestyle factors therefore cannot be fully excluded. Our associations remain hypothesis-generating rather than deconfounded causal effects.

In summary, this study provides an integrated functional metagenomic and metabolic characterization of the gut microbiome across LST histological subtypes and grades. It suggests that HGIN, relative to LGIN and SSL, is associated with enrichment for capacities related to inflammation, antibiotic resistance, and mobile genetic elements, potentially providing a basis for future mechanistic studies and the development of microbial biomarkers associated with high-grade histopathology within the LST spectrum.

## Data Availability

The data presented in the study are deposited in the GSA-Human database (https://ngdc.cncb.ac.cn/gsa-human/), accession number HRA018964.
